# Predicting Adherence to Behavior Change Support Systems Using Machine Learning: Systematic Review

**DOI:** 10.2196/46779

**Published:** 2023-11-22

**Authors:** Akon Obu Ekpezu, Isaac Wiafe, Harri Oinas-Kukkonen

**Affiliations:** 1 Oulu Advanced Research on Service and Information Systems Department of Information Processing Science University of Oulu Oulu Finland; 2 Department of Computer Science University of Ghana Accra Ghana

**Keywords:** adherence, compliance, behavior change support systems, persuasive systems, persuasive technology, machine learning

## Abstract

**Background:**

There is a dearth of knowledge on reliable adherence prediction measures in behavior change support systems (BCSSs). Existing reviews have predominately focused on self-reporting measures of adherence. These measures are susceptible to overestimation or underestimation of adherence behavior.

**Objective:**

This systematic review seeks to identify and summarize trends in the use of machine learning approaches to predict adherence to BCSSs.

**Methods:**

Systematic literature searches were conducted in the Scopus and PubMed electronic databases between January 2011 and August 2022. The initial search retrieved 2182 journal papers, but only 11 of these papers were eligible for this review.

**Results:**

A total of 4 categories of adherence problems in BCSSs were identified: adherence to digital cognitive and behavioral interventions, medication adherence, physical activity adherence, and diet adherence. The use of machine learning techniques for real-time adherence prediction in BCSSs is gaining research attention. A total of 13 unique supervised learning techniques were identified and the majority of them were traditional machine learning techniques (eg, support vector machine). Long short-term memory, multilayer perception, and ensemble learning are currently the only advanced learning techniques. Despite the heterogeneity in the feature selection approaches, most prediction models achieved good classification accuracies. This indicates that the features or predictors used were a good representation of the adherence problem.

**Conclusions:**

Using machine learning algorithms to predict the adherence behavior of a BCSS user can facilitate the reinforcement of adherence behavior. This can be achieved by developing intelligent BCSSs that can provide users with more personalized, tailored, and timely suggestions.

## Introduction

Behavior change support systems (BCSSs) have been effective in improving health and healthier lifestyles. These are persuasive systems that have been designed to change behavior without force or deception [1]. However, the effectiveness of these systems is generally hindered by nonadherence [2-4]. Nonadherence to recommended regimes in BCSSs has the potential to diminish their long-term benefits [5]. It is associated with the increased prevalence of diseases such as hypertension, diabetes, obesity, dementia, bipolar disorder, and heart failure [2,4,6-8], as well as the increased cost of health care. Yet, there are no standardized factors that can reliably predict adherence [9,10]. Direct adherence monitoring approaches are expensive, burdensome to care providers, and susceptible to distortion by patients, while indirect monitoring approaches such as pill count, patient questionnaires, electronic medication monitors, or electronic reporting of daily physical activity are susceptible to misinterpretations and overestimation of adherence [11,12]. To implement effective BCSSs and ensure positive behavior change outcomes that can be attributed to the recommended interventions, an accurate assessment of adherence behaviors and their predictors has become imperative. This will guide researchers and health care providers in identifying nonadherent individuals as well as provide measures that will re-engage and help them to adhere [13]. Additionally, an early prediction of user dropout or relapse during interventions may suggest measures that can be used to improve adherence [14].

Existing systematic reviews [2,7,15-19] have sought to examine predictors or determinants of adherence to several BCSSs. They predominately report that there is a lack of consistency regarding reports of adherence, key variables mediating adherence, and reliable measures of adherence. However, findings from these reviews were based on studies that relied solely on self-reported measures of adherence using pharmacological claims and validated questionnaires from behavior change and health psychology theories. Hence, abounding issues of over- and underreporting may limit the validity of the findings.

This review enhances existing knowledge by focusing on predictors of adherence to BCSSs using machine learning techniques. Machine learning techniques have enabled a proficient means of classifying, detecting, and predicting complex phenomena including human behavior. It has also attracted considerable research interest in the development of BCSSs [20-22]. Nonetheless, literature on the use of machine learning techniques as adherence prediction methods in BCSSs is limited [13,23]. Although Bohlmann et al [23] provided literature summaries on machine learning techniques for predicting adherence, they focused on medication adherence only and considered both digital and nondigital interventions. In contrast to previous reviews, this systematic review focuses on the use of machine learning approaches to predict all kinds of adherence problems in BCSSs. In addition, it focuses only on primary studies that used objectively collected data or data generated by the BCSS. Accordingly, this review seeks to answer the following question: What are the existing trends in the use of machine learning techniques to predict adherence to BCSSs? Specifically, this study answers 4 main review questions, as shown in Table 1.

**Table 1:** Review questions (RQs) and their motivations.

RQ	Question	Motivation
RQ1	What are the targeted adherence problems and their related definitions?	Research on adherence has predominately focused on adherence to medication and pharmacological treatments. However, adherence covers a wider range of health behaviors than medication adherence [9]. This RQ sought to identify other target adherence problems in BCSSs^a^.
RQ2	What are the characteristics of the BCSS including persuasive system features?	Considering the variabilities in adherence problems and BCSSs, this RQ aimed to provide summaries on the characteristics of the BCSS and the persuasive system features that have been used to improve adherence.
RQ3	What are the adopted machine learning approaches in predicting adherence to BCSSs?	This RQ sought to identify the nature of the raw data and predominately used machine learning techniques, feature selection techniques, and performance metrics.
RQ4	What are the limitations or barriers to adherence?	Though various barriers to adherence have been identified in the literature, this RQ sought to identify only those barriers that limit individuals from adhering to the request of the BCSS.

^a^BCSS: behavior change support system.

## Methods

### Literature Search

Following the Preferred Reporting Items for Systematic Reviews and Meta-Analyses (PRISMA) approach, a search on Scopus and PubMed electronic databases was conducted. This search aimed to identify peer-reviewed English conference and journal papers published between January 2011 and August 2022. Scopus indexes a larger number of peer-reviewed scientific journals than the Web of Science and offers results of more consistent accuracy than Google Scholar, while PubMed remains a leading database in biomedical research [24]. Including papers published within the past decade will reveal recent evidence-based research trends [25]. Using the logical OR/AND operators, the search phrases were a combination of keywords related to prediction, adherence, health behavior change interventions, and machine learning (See Multimedia Appendix 1 for the search phrases). Considering the plethora of approaches to investigate adherence, the search for eligible studies was not limited to a specific study design.

Only empirical studies that described the development and testing of machine learning models for BCSS adherence prediction were considered. Studies that used only self-reported data, were not reported in English, or did not focus on human participants were ignored.

### Study Selection

During the initial search of the databases, 2182 papers were retrieved. The results were refined by year, document type, publication stage, source type, and language, resulting in 1866 papers. The exported papers were screened for uniqueness and for titles containing keywords such as adherence, prediction, and any machine learning technique. Of these, 1812 papers were excluded. The remaining 54 papers were screened by abstracts and full texts. Papers were excluded by abstract if the machine learning technique(s) were not mentioned. Furthermore, papers were excluded by full text if the intervention was not characterized by a BCSS (any form of information system that has been developed to change human behavior voluntarily). Finally, 11 journal papers were considered eligible for this systematic review and thus downloaded for methodological quality assessment.

**Figure 1:**
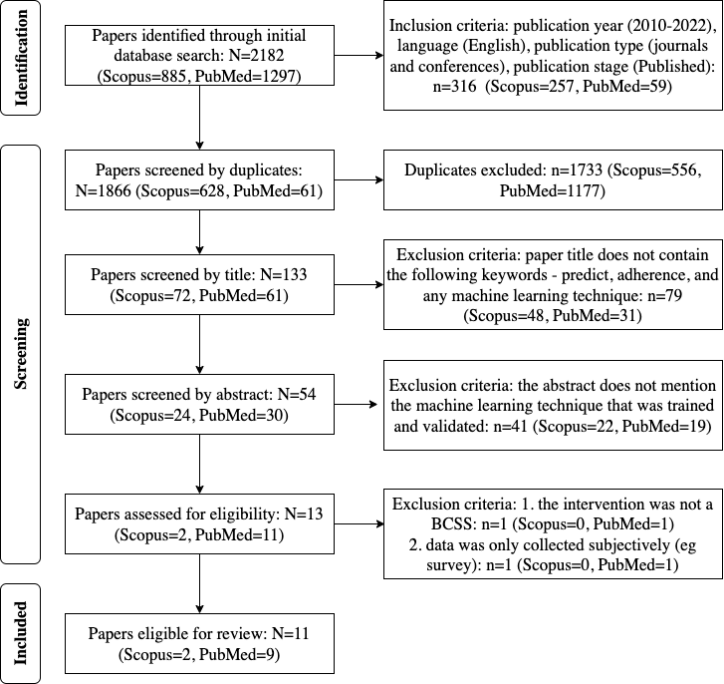
PRISMA (Preferred Reporting Items for Systematic Reviews and Meta-Analyses) flow diagram for study selection. BCSS: behavior change support systems.

Figure 1 shows the PRISMA flow diagram of study identification and selection. Since the study selection was not limited to a specific study design, the Mixed Methods Appraisal Tool by [26] was used to assess the methodological quality of the downloaded papers (see Multimedia Appendix 2 [13,14,26-35]). Accordingly, 11 studies were identified to be of high methodological quality. Pertinent information on the study characteristics and machine learning approaches was extracted using a data extraction form in Microsoft Excel created by the authors. Multimedia Appendix 3 [13,14,27-35] presents a list of included studies.

## Results

All included studies (N=11) were primarily aimed at the development and use of machine learning techniques for the prediction of adherence [27-32], nonadherence, dropout, or relapse [13,14,33-35]. The ensuing sections will elaborate on findings related to the review questions (RQs).

### Targeted Adherence Problems and Their Related Definitions (RQ1)

#### Overview

This review identified 7 health behaviors that BCSSs target: medication adherence [29,31,34], use of health care systems [13,28], physical activity [14,27], diet [33], illicit drug use [30], depression and anxiety [32], and insomnia [35]. Based on the characteristics of these health behaviors, they were grouped into 4 categories of adherence problems, described in the following sections.

#### Adherence to Digital Cognitive or Behavioral Interventions (n=5)

This category includes health behaviors such as health care system use, illicit drug use, depression, anxiety, and insomnia. This adherence problem focused on predicting adherence or nonadherence to internet-based cognitive behavioral therapy [32,35], and remote health monitoring systems [13,28,30] using machine learning models. Adherence to digital cognitive or behavioral interventions refers to the successful completion of all recommended tasks and achievement of initial set goals. While nonadherence refers to ignoring or not completing the recommended task consecutively after signing up to use the BCSS.

#### Medication Adherence (n=3)

This adherence problem is linked to medication adherence behavior. Although extant literature posit that medication nonadherence is the most common form of adherence problem, this review identified 3 studies that addressed this problem within BCSSs. It compromises the effectiveness of treatment outcomes in about 85% of patients with chronic and acute medical conditions globally [11,36]. Studies in this category applied machine learning models to remote real-time measurements of medication dosing [29,31,34]. Though these studies had different thresholds for defining medication adherence, it generally referred to a patient’s behavior or commitment to taking the medications as prescribed by a physician with an average adherence rate of 80% and above.

#### Physical Activity Adherence (n=2)

Adhering to physical activity routines has the potential to reduce the risk of chronic diseases irrespective of age or other sociodemographic factors. Whereas some individuals find it difficult to regularly engage in or continue a physical activity routine [14], others discontinue when they have achieved a health or body goal [27]. These studies were observed to have varying definitions of adherence. For instance, Zhou et al [14] considered an increase in the number of steps over time, while Bastidas et al [27] considered the users’ app use patterns. Thus, physical activity adherence may be defined as either a consistent increase in physical activity levels compared to an individual’s baseline activity levels or an individual’s responsiveness to prompts from the app. These 2 definitions describe behavior compliance and program compliance, respectively [37].

#### Diet Adherence (n=1)

This was observed in only 1 study [33]. Dietary relapse in a weight loss intervention was predicted. Dietary relapse refers to any instance in which a person exceeded a specified meal or snack point threshold (per meal).

### Characteristics of the BCSSs and Persuasive System Features (RQ2)

The BCSSs included mobile apps [13,14,27,28,33,34], web-based apps [30,32,33,35], and sensor-based systems plus mobile apps [29,31]. They were targeted at different groups of people, namely physically inactive women, illicit drug users, obese or overweight people, and patients with a wide range of chronic diseases, such as heart failure, myocardial infarction-anxiety, depression (MI-ANXDEP), insomnia, and Parkinson disease. Multimedia Appendix 4 [14,15,29-44] describes other study-specific characteristics.

The BCSSs leveraged some behavior change techniques and persuasive systems features to improve user adherence. These features were extracted and evaluated using the persuasive systems design (PSD) model [45]. The PSD model has been validated in several studies [37] and is predominately used in the design and evaluation of BCSSs [46]. The model consists of 28 system features that make up 4 categories of persuasive principles (namely primary task support, dialogue support, credibility support, and social support). Table 2 displays the frequency of the PSD features represented in the BCSS. All the studies had primary task support features, 8 studies had dialogue support features, 5 studies had credibility support features, and only 1 study had social support features.

**Table 2:** Persuasive features identified in the behavior change support systems (BCSSs). Check marks indicate that the feature was identified.

Persuasive features		Studies predicting adherence							Studies predicting nonadherence						Total
		[30]	[28]	[32]	[29]	[31]	[27]	[13]		[35]	[33]	[34]	[14]		
**Primary task support**															
	Personalization	✓		✓	✓	✓	✓	✓		✓		✓	✓	8/11	
	Self-monitoring		✓				✓	✓					✓	4/11	
	Reduction				✓		✓				✓	✓		4/11	
	Rehearsal					✓								1/11	
	Tailoring	✓												1/11	
**Dialogue support**															
	Reminders		✓		✓					✓	✓	✓		5/11	
	Suggestions							✓					✓	2/11	
	Praise	✓											✓	2/11	
	Rewards										✓			1/11	
	Similarity	✓												1/11	
	Liking									✓				1/11	
**Credibility support**															
	Real world feel	✓					✓			✓		✓	✓	5/11	
	Expertise	✓					✓			✓				3/11	
	Verifiability	✓								✓		✓		3/11	
	Third party endorsement	✓												1/11	
**Social support**															
	Social facilitation	✓						✓						2/11	

Primary task support simplifies and motivates users to perform recommended tasks (eg, exercise). A total of 5 features of the primary task support principle were used: personalization, self-monitoring, reduction, rehearsals, and tailoring. Personalization was the most used feature in this category. It delivers personalized content to the users. For example, artificial intelligence generated workouts according to an individual’s characteristics and preferences. Self-monitoring enables app users to view and track their activity levels and health status in real-time (eg, the app enables users to monitor, visualize, and track activity levels and calories burned in real time). Reduction breaks down tasks such as daily point goals into specific meal or snack targets. The least used features in this category were rehearsal (practicing the target behavior, eg, gait movements) and tailoring (eg, the app provided content that was distinct to users of specific age groups and health goals, such as alcohol or smoking cessation, weight loss, or mental health).

Dialogue support provides a means to help users to achieve their goals via human-computer interactions. The dialogue support features included reminders (eg, medication prompts), suggestions (eg, the app advises users based on their input to the app), and praise (automated feedback on the completion of a task). The least used features included rewards (eg, point-based incentives), similarity (eg, therapy resembling traditional cognitive behavioral therapy), and liking (eg, user-friendly and appealing design).

System credibility support provides a means for users to trust the system. Features identified in this category included expertise (app provided theory-based information and were designed to improve engagement, effectiveness, and security), verifiability (app provided links to related sites), third-party endorsement (from the National Institute of Health), and real-world feel. The real-world feel feature was implemented as “Contact Us” (a means to communicate with the developers of the app) and “About Us” (providing information about the developers of the app).

Social support provides a means of supporting users via social influence. However, it was the least used principle. Social facilitation was the only identified social support feature and it was implemented by allowing user participation in online app forums. Perhaps, the minimal use of social support features may be attributed to the negative sentiments associated with some of its features [38].

It is important to note that the effectiveness of these persuasive system features in improving adherence was not explicitly evaluated in the included studies. However, these features may have directly or indirectly improved adherence rates. Some of the studies used behavior change techniques such as goal-setting, web-based human coaching, face-to-face counseling sessions, and feedback from psychologists and expert program providers to improve adherence behavior. However, this review could not identify behavioral theories or models upon which these techniques were based.

### Machine Learning Approaches (RQ3)

Developing machine learning models for predicting user adherence or nonadherence was the general aim of all the included studies. This process conventionally consists of 4 main stages: data collection, feature selection, model training, and model validation.

### Data Collection

Apart from the gait-related data, which were collected in a controlled environment (laboratory), the data used to train or test the machine learning models in the majority of the studies were objectively collected by the health app while the study participants were performing the behavior of interest in an uncontrolled environment. For example, data such as log data, training behavior, walking steps, and 3D movement scans were automatically extracted in a contactless manner without any form of self-report from the users.

Studies on medication adherence were observed to use images and videos to capture participants’ medication adherence behavior. The apps used technologies such as computer vision [34], internet-connected smart sharp bin [29], and flight sensors [31] for the real-time monitoring of self-administered injections and medication ingestion. Time-stamped data of injection needles discarded into the smart sharp bin, time-stamped skeletal joint data, and images of the participants taking the medication were retrieved, validated, and then used to generate the data set for training or testing the model. Similarly, studies on physical activity adherence [14,27] used continuously collected time-dependent physical activity data from app users to develop machine learning models.

Studies on adherence to digital cognitive or behavioral interventions [13,28,30,32,35] and dietary adherence [33] used a combination of objectively collected data and participants’ responses to questions on self-assessment delivered by the health app. Goldstein et al [33] used a BCSS that asked predefined questions related to triggers of dietary relapse for the analysis. Considering that objectively collecting trigger-related data or self-assessment data on health symptoms from a mobile app may currently be challenging as triggers (such as food cravings and hunger) are physiologically motivated, future studies on BCSSs that seek to extract trigger-related data may consider using physiological sensor data. This is a noninvasive approach to detecting hunger and cravings using wearable body sensors [39]. Such sensor data may also be integrated into the health app to enable self-monitoring.

### Feature Selection or Engineering

Among the 11 studies, 5 performed feature selection, 5 performed feature engineering, and 1 adopted features based on existing literature (see Multimedia Appendix 5 [14,15,29-37]). Feature selection and feature engineering were both aimed at enhancing model performance by eliminating irrelevant features and generating new features from raw data respectively. Due to the complexities of combining 2 or more machine learning techniques (ie, ensemble learning), some studies [28] applied more than 1 feature selection method. However, there were no differences in the selected features.

The flat features algorithm (including filter, wrapper, and embedded methods) were the predominately used feature selection method. This algorithm assumes all features to be independent [40]. Interestingly, each study had its own set of unique predictors irrespective of the category of adherence problem. Multimedia Appendix 5 [14,15,29-37] highlights the various feature selection approaches.

### Model Training

The learning problem was a binary classification. Thus, there were 2 class labels (outcomes), namely adherence/nonadherence, adherers/nonadherers, relapse/nonrelapse, and dropout/nondropout. An overview of the adopted techniques and the outcomes of the best-performing techniques is provided in Table 3.

**Table 3:** Identified machine learning and model validation techniques.

Ref	Machine learning techniques	Evaluation metrics	Predicted outcome
[30]	Logistic regression and random forest^a^	AUROC^a,b^, specificity, sensitivity, PPV^c^, NPV^d^, and confusion matrix	Successful or early dropout
[13]	Logistic regression, random forest^a^, and decision trees	Accuracy, precision^a^, and AUROC	Dropout or nondropout
[28]	Decision tree, MLP^e^, and KNN^a,f^	Precision, sensitivity, *F*_1_-score, TPR^g^, FPR^h^, and AUROC^a^	Adherers or nonadherers
[32]	Random forest^a^	Accuracy	Adherence or nonadherence
[35]	Logistic regression, SVM^i^, and decision trees (boosted)^a^	AUROC^a^, TPR, FPR, and PRAUC^j^	Dropout or nondropout
[33]	Ensemble methods^a^	Accuracy, sensitivity^a^, specificity, and AUROC^a^	Relapse or not
[29]	XGB^k^, extra trees, random forest, MLP, gradient tree boosting, recurrent neural network, and LSTM^a,l^	Accuracy, specificity^a^, sensitivity, precision, *F*_1_-score, and AUROC	Adherence or nonadherence
[34]	XGB^a^	Accuracy, precision^a^, sensitivity, AUROC, TPR, and FPR	Adherence or nonadherence
[31]	Decision trees^a^, KNN, naive Bayes, SVM, and random forest	Confusion matrix	Adherence or nonadherence
[27]	LSTM^a^ and SVM	Accuracy, sensitivity, *F*_1_-score, and confusion matrix	Adherent or nonadherent
[14]	Logistic regression^a^ and SVM	Accuracy, sensitivity, specificity, and AUROC	Relapse or not

^a^Best performing machine learning technique or most relevant metric for the outcome prediction.

^b^AUROC: area under the receiver operating characteristic curve.

^c^PPV: positive predictive value.

^d^NPV: negative predictive value.

^e^MLP: multilayer perceptron.

^f^KNN: k-nearest neighbor.

^g^TPR: true positive rate.

^h^FPR: false positive rate.

^i^SVM: support vector machine.

^j^PRAUC: precision-recall curve.

^k^XGB: extreme gradient boost.

^l^LSTM: long short-term memory.

A total of 13 supervised machine-learning techniques were used across the included studies. Logistic regression, support vector machines, and random forest were the most used techniques cutting across all 4 categories of the adherence problems. The machine learning techniques mapped to specific adherence problems included support vector machines for physical activity adherence; extreme gradient boosting, extra trees, recurrent neural network, naive Bayes, and gradient tree boosting for medication adherence; and ensemble methods for dietary adherence. Random forest was observed to be the predominant best-performing model in studies on adherence to digital cognitive or behavioral interventions, while long short-term memory (LSTM) was a common best-performing model between medication adherence and physical activity adherence. Overall, the predominant best-performing models across all included studies were random forest, decision trees, logistic regression, k-nearest neighbor, LSTM, and ensemble learning.

### Model Validation

Owing to the relatively small and imbalanced data sets used in some of the included studies, cross-validation methods were adopted to eliminate bias that may occur during data split. The following cross-validation methods were identified: K(5)-fold cross-validation [29,34]; leave-one-out cross-validation [28,31,33]; and stratified K(10)-fold cross-validation [13,30,35].

Besides cross-validation methods, several performance metrics were used to compare and evaluate the performance of the various machine learning models. It was observed that the choice of performance metrics was dependent on the context of the study and more than 1 metric was used to evaluate the performance of a model (see Table 3). The predominately used metrics in order of frequency included area under the receiver operating characteristic curve (7/11), accuracy (6/11), sensitivity (6/11), specificity (4/11), precision (3/11), *F*_1_-score (3/11), true positive rate (3/11), false positive rate (3/11), confusion matrix (3/11), positive predictive value (1/11), negative predictive value (1/11), and precision-recall curve (1/11).

Generally, it was observed that the prediction models had good classification accuracies. This was an indication that the features or predictors used in each of these studies were a good representation of the intervention domain. Nonetheless, due to the plethora of digital platforms, the interaction between technology and behavior may affect the generalizability of the results [33,34]. Thus, Koesmahargyo et al [34] posit replication and integration of data from various digital platforms.

### Barriers to Adherence (RQ4)

Studies suggest that the rate of adherence may be affected by the following:

1. Achievement of set health or body goals
2. Issues associated with trust and the tolerability of the technology
3. The complexity of the system, and the mismatch between the system design and the needs and preferences of its users
4. Inappropriate timing for delivering or sending notifications or suggestions to the users; since these timings are usually not well chosen, they may either inconvenience the users when delivered or may not be effective in getting their attention
5. The insufficient open collaborative relationship between health app providers and the users. Prior studies [37] refer to this as a lack of accountability in adherence prediction models

These barriers may be classified into two nonadherent groups: intentional nonadherence (1 and 2), or unintentional nonadherence (3, 4, and 5).

## Discussion

This systematic review provides an overview of existing trends in the use of machine learning techniques to predict adherence to different BCSSs. This was achieved by finding answers to a set of review questions using data extracted from the 11 included studies. The rest of this section will summarize and discuss findings based on the review results.

This review identified 4 categories of adherence problems: adherence to digital cognitive or behavioral interventions, medication adherence, physical activity adherence, and diet adherence. These problems collectively represent what Middleton et al [4] describe as an “adherence challenge.” However, when considering the taxonomy of key health behaviors [41], it was observed that the behaviors identified in this systematic review were not exhaustive. Consequently, the prediction of adherence to other health behaviors is an open research area for further investigation.

On the use of persuasive system design features, it was observed that primary task support features were the most used, while social support features were the least used. This finding is consistent with that of related systematic reviews [37]. Though the included studies claimed that either the implementation of the BCSS or the selected features (predictors) for the machine learning algorithm was theory-based, this systematic review could not identify the behavior change theories adopted by the studies. Hence, it was not clear if the operating mechanisms of behavioral theories were considered in most of the included studies. Nonetheless, prior studies have provided evidence of the effectiveness of theory-based interventions and persuasive system features in promoting adherence behavior in BCSSs. Future studies should therefore be intentional about the use of these mechanisms as measures of improving adherence behavior.

The relevant predictors identified align with findings from existing literature. Similar to existing reviews [4,42], exercise history, intensity, and frequency emerged as relevant predictors of physical activity adherence. Exercise, fatigue, cognitive load, and confidence were the most relevant predictors of diet adherence, affirming previous findings (eg, [42]). While communication with or feedback/advice from the physician or health provider, fear, and patients’ cognitive capacity were the most relevant predictors of medication adherence as also found in past reviews (eg, [3,42]). Furthermore, some of the identified predictors of physical activity adherence and medication adherence affirm 2 viewpoints from social learning theory [43]: that individuals develop beliefs that they can perform the necessary tasks to obtain the desired outcome based on prior accomplishment of similar behaviors and verbal persuasion by credible sources. This systematic review identified the completion of homework assignments as a predictor of cognitive or behavioral intervention adherence, while Heesch et al [44] identified the same predictor for physical activity adherence. Furthermore, this review suggests that not all initially selected predictors or features of adherence are subsequently considered most relevant by the feature selection algorithms (see Multimedia Appendix 5 [14,15,29-37]). Using multiple feature selection methods yields the same feature set. Future studies should consider incorporating the feature selection or engineering techniques identified in this review to enable a comparison of their results with the existing literature.

Most of the included studies used traditional machine learning techniques, with limited use of advanced learning techniques such as ensemble learning, reinforcement learning, and deep learning. Among the 13 supervised machine learning algorithms, only 2 were deep learning techniques (multilayer perceptron and recurrent neural network–LSTM), 1 ensemble, and zero reinforcement learning. Perhaps the sparing use of deep learning techniques may be attributed to the small sample sizes of these studies, considering that deep learning is more efficient in the analysis of huge amounts of data. For instance, LSTM may have been a more appropriate algorithm for the study by Evangelista et al [28], because the data collected captured changes in conditions that evolved slowly over time. However, it was not used probably due to a sample of only 14 participants. Interestingly, in a study with a large data set (342,174 injection historic drop data) [29], LSTM outperformed traditional machine learning models like random forest. Future studies should therefore consider using advanced learning methods instead of traditional learning techniques. Deep learning techniques can automate feature engineering or selection and extract complex and nonlinear patterns from data. Reinforcement learning is well suited for systems with inherent time delays where decisions are evaluated by a long-term future reward and not an immediate knowledge of the effectiveness of a system [47]. In addition, since they learn by observing the results of their actions, they are applicable in study settings with scarce or varying data as found in BCSSs [22,47].

This systematic review observed that quite a small amount of data were used in most of the included studies. With each study participant treated as a single data point, data were extracted from as little as 12 study participants to as many as 7697 study participants. Although training a machine learning model requires a reasonable amount of data to train the model, the required sample size for training and producing a model with good generalizability is not well established [33,48]. Regardless, the included studies adopted suitable machine learning techniques, dimensionality reduction techniques, and evaluation methods that are designed to improve model performance irrespective of the sample size. For instance, Zhou et al [14] performed data augmentation on the training data.

Multiple metrics can be used to evaluate model performance (see Table 3). However, the choice of which metrics best measure the model performance depends on the nature of the problem, the researcher’s understanding of the domain or problem, and the expected outcome of the study. For instance, Gu et al [29] prioritized predicting nonadherers (those who will not perform the recommended task on time), hence specificity (true negative rate) was a preferred metric. Considering that a wrong prediction may lead the health app provider to develop unnecessary persuasive strategies for the user, Pedersen et al [13] and Bastidas et al [27] aimed to reduce false negatives (ie, participants at high risk of dropout are not identified as such), hence a high precision was a preferred metric. However, if the study’s goal is to validate the hypothesis that machine learning methods can be used in predicting adherence [14,31] rather than to compare machine learning methods, then choosing the most appropriate metric becomes irrelevant.

A major research challenge reported in 9 of 11 of the included studies was the scarce and small-sized data sets and their effect on the generalizability and reliability of the research results. A specific study limitation pertained to collecting data in a controlled environment [31]. This method of data collection does not represent the entire range of user behavior in a free-living environment.

### Conclusions

Findings from this systematic review indicate that though the use of machine learning techniques in the prediction of adherence to BCSSs is scarce and is only beginning to gain research interest, it has the potential to accurately predict adherence behavior in real time using objectively collected data. This systematic review is unique as it has not yet been reported in the literature, and it provides an overview of machine learning approaches in determining predictors of specific adherence problems in BCSSs. A grasp of these trends across different BCSSs will guide researchers in choosing appropriate features and machine learning techniques that favor the prediction of specific adherence problems. In summarizing findings from 11 journal papers, this systematic review highlights research gaps and areas for future research. It also acknowledges limitations that may exist due to the selection strategy for eligible studies.

## Appendix

Multimedia Appendix 1 Search syntax and results.

Multimedia Appendix 2 Multimedia quality assessment.

Multimedia Appendix 3 List of reviewed primary studies.

Multimedia Appendix 4 Characteristics of the studies and the BCSSs.

Multimedia Appendix 5 Input data and feature selection approaches.

## Abbreviations

BCSS: behavior change support system
LSTM: long short-term memory
MI-ANXDEP: myocardial infarction-anxiety, depression, or both
PSD: persuasive systems design
PRISMA: Preferred Reporting Items for Systematic Reviews and Meta-Analyses
RQ: review question
