# Supplementary material for: Predicting Adherence to Behavior Change Support Systems Using Machine Learning: Systematic Review
Source: JMIR AI. 2023 Nov 22;2:e46779. doi: 10.2196/46779 (PMC11041458; doi:10.2196/46779)
Supplement: Multimedia Appendix 1 [file ai_v2i1e46779_app1.docx]

# APPENDIX 1. SEARCH SYNTAX AND STUDY SELECTION

| DATABSES |  | SEARCH PHRASES | Initial search results | Exclude by year, language pub type, pub stage | Remove duplicate within database | Remove duplicate between databases | Exclude by title | Exclude by abstract | Exclude by full-text |
| --- | --- | --- | --- | --- | --- | --- | --- | --- | --- |
| SCOPUS | SP1 | TITLE-ABS-KEY ((predict* OR determin* OR classifi* OR detect* ) AND (*adherence OR *compliance OR relapse OR dropout OR attrition OR *engagement) AND ("smoking cessation" OR smok* OR alcohol OR "physical activity" OR exercise OR nutrition OR diet* OR health* OR lifestyle OR behaviour* OR behavior*) AND (intervention* OR program* OR treatment) AND ("machine learning" OR "deep learning" OR "reinforcement learning" OR "neural network*" OR "ensemble learning")) | 394 | 328 | 96 | 72 | 24 | 2 | 2 |
|  | SP2 | TITLE-ABS-KEY ((predict* OR determin* OR classifi* OR detect*) AND (adherence OR compliance OR non-adherence OR non-compliance OR relapse OR dropout OR attrition) AND ("digital health" OR mHealth OR eHealth OR "mobile health" OR "electronic health" OR web* OR smartphone OR online* OR internet*) AND (intervention* OR program* OR treatment) AND ("machine learning" OR "deep learning" OR "reinforcement learning" OR "neural network*" OR "ensemble learning")) | 180 | 143 |  |  |  |  |  |
|  | SP3 | TITLE-ABS-KEY (("machine learning" OR "deep learning" OR "reinforcement learning" OR "neural network*" OR "ensemble learning") AND (adherence OR compliance OR "non-adherence" OR "non-compliance") AND (predict)) | 311 | 157 |  |  |  |  |  |
| PUBMED | SP1 | (predict* OR determin* OR classifi* OR detect*) AND (adherence OR compliance OR non-adherence OR non-compliance OR relapse OR dropout OR attrition) AND ("smoking cessation" OR smok* OR alcohol OR "physical activity" OR exercise OR nutrition OR diet* OR health* OR lifestyle OR behaviour* OR behavior*) AND (intervention* OR program* OR treatment) AND ("machine learning" OR "deep learning" OR "reinforcement learning" OR "neural network*" OR "ensemble learning") | 837 | 790 | 91 | 61 | 30 | 11 | 9 |
|  | SP2 | (predictors OR predict OR determinants OR classification OR detect) AND (adherence OR compliance OR relapse OR dropout OR attrition OR engagement) AND ("digital health" OR mHealth OR eHealth OR "mobile health" OR "electronic health" OR web* OR smartphone OR online* OR internet*) AND (intervention* OR program* OR treatment) AND ("machine learning" OR "deep learning" OR "reinforcement learning" OR "neural network*" OR "ensemble learning") | 344 | 337 |  |  |  |  |  |
|  | SP3 | (“machine learning” OR “deep learning” OR “reinforcement learning” OR “neural network*” OR “ensemble learning”) AND (adherence OR compliance OR “non-adherence” OR “non-compliance”) AND predict | 116 | 111 |  |  |  |  |  |
|  |  | Totals | 2182 | 1866 | 187 | 133 | 54 | 13 | 11 |
