# Supplementary material for: Predicting Adherence to Behavior Change Support Systems Using Machine Learning: Systematic Review
Source: JMIR AI. 2023 Nov 22;2:e46779. doi: 10.2196/46779 (PMC11041458; doi:10.2196/46779)
Supplement: Multimedia Appendix 2 [file ai_v2i1e46779_app2.docx]

**METHODOLOGICAL QUALITY ASSESSMENT**

The mixed methods appraisal tool (MMAT) checklist for non-randomized studies (Hong et al [26]) was adapted. Studies were classified as high (6-7 items met); moderate (4-5 items met); and low (less than 3 items met) quality based on the outcome of questions. However, no studies were excluded based on methodological quality.

| Ref No | First author | Year | Are there clear research questions? | Do the collected data allow to address the research questions? | Are the participants representative of the target population? | Are measurements appropriate regarding both the outcome and intervention (or exposure)? | Are there complete outcome data? | Are the confounders accounted for in the design and analysis? | During the study period, is the intervention administered (or exposure occurred) as intended? | Comments |
| --- | --- | --- | --- | --- | --- | --- | --- | --- | --- | --- |
| [30] | Ramos, L A | 2021 | Yes | Yes | Yes | Yes | Yes | Can’t tell | Yes | 6/7 - High |
| [13] | Pedersen, D H | 2019 | Yes | Yes | Yes | Yes | No | Can’t tell | Yes | 6/7 - High |
| [28] | Evangelista, L S | 2017 | Yes | Yes | Yes | Yes | Yes | Can’t tell | Yes | 6/7 - High |
| [32] | Wallert, J | 2018 | Yes | Yes | Yes | Yes | Yes | Can’t tell | Yes | 6/7 - High |
| [35] | Bremer, V | 2019 | Yes | Yes | Yes | Yes | Yes | Can’t tell | Yes | 6/7 - High |
| [33] | Goldstein, S P | 2019 | Yes | Yes | Yes | Yes | Yes | Can’t tell | Yes | 6/7 - High |
| [29] | Gu, Y | 2021 | Yes | Yes | Yes | Yes | Yes | Can’t tell | Yes | 6/7 - High |
| [34] | Koesmahargyo, V | 2020 | Yes | Yes | Yes | Yes | Yes | Can’t tell | Yes | 6/7 - High |
| [31] | Tucker, C | 2015 | Yes | Yes | Yes | Yes | Yes | Can’t tell | Yes | 6/7 - High |
| [27] | Bastidas, O J | 2021 | Yes | Yes | Yes | Yes | Yes | Can’t tell | Yes | 6/7 - High |
| [14] | Zhou, M | 2019 | Yes | Yes | Yes | Yes | Yes | Yes | Yes | 7/7 - High |
