# Supplementary material for: Predicting Adherence to Behavior Change Support Systems Using Machine Learning: Systematic Review
Source: JMIR AI. 2023 Nov 22;2:e46779. doi: 10.2196/46779 (PMC11041458; doi:10.2196/46779)
Supplement: Multimedia Appendix 3 [file ai_v2i1e46779_app3.docx]

**Appendix 3.** List of the 11 reviewed studies

| Ref No | Reference |
| --- | --- |
| [30] | Ramos, L A, Blankers, M, van Wingen, G, de Bruijn, T, Pauws, S C, & Goudriaan, A E (2021). Predicting Success of a Digital Self-Help Intervention for Alcohol and Substance Use With Machine Learning. *Frontiers in Psychology*, *12*(September), 1-11. https://doi.org/10.3389/fpsyg.2021.734633 |
| [13] | Pedersen, D H, Mansourvar, M, Sortsø, C, & Schmidt, T (2019). Predicting dropouts from an electronic health platform for lifestyle interventions: Analysis of methods and predictors. *Journal of Medical Internet Research*, *21*(9), 1-12. https://doi.org/10.2196/13617 |
| [28] | Evangelista, L S, Irvine, C, Ghasemzadeh, H, Science, C, State, W, Lee, J, Fallahzadeh, R, Science, C, State, W, Sarrafzadeh, M, Angeles, L, Moser, D K, & Endowed, G (2017). Predicting Adherence to Use of Remote Health Monitoring Systems in a Cohort of Patients with Chronic Heart Failure. *Technology Health Care*, *25*(3), 425-433. https://doi.org/10.3233/THC-161279. |
| [32] | Wallert, J, Gustafson, E, Held, C, Madison, G, Norlund, F, Von Essen, L, & Olsson, E M G (2018). Predicting adherence to internet-Delivered psychotherapy for symptoms of depression and anxiety after myocardial infarction: Machine learning insights from the U-CARE heart randomized controlled trial. *Journal of Medical Internet Research*, *20*(10). https://doi.org/10.2196/10754 |
| [35] | Bremer, V, Chow, P I, Funk, B, Thorndike, F P, & Ritterband, L M (2020). Developing a Process for the Analysis of User Journeys and the Prediction of Dropout in Digital Health Interventions: Machine Learning Approach. *Journal of Medical Internet Research*, *22*(10). https://doi.org/10.2196/17738 |
| [33] | Goldstein, S P, Zhang, F, Thomas, J G, Butryn, M L, Herbert, J D, & Forman, E M (2018). Application of Machine Learning to Predict Dietary Lapses During Weight Loss. *Journal of Diabetes Science and Technology*, *12*(5), 1045-1052. https://doi.org/10.1177/1932296818775757 |
| [29] | Gu, Y, Zalkikar, A, Liu, M, Kelly, L, Hall, A, Daly, K, & Ward, T (2021). Predicting medication adherence using ensemble learning and deep learning models with large-scale healthcare data. *Scientific Reports*, *11*(1), 1-13. https://doi.org/10.1038/s41598-021-98387-w |
| [34] | Koesmahargyo, V, Abbas, A, Zhang, L, Guan, L, Feng, S, Yadav, V, & Galatzer-Levy, I R (2020). Accuracy of machine learning-based prediction of medication adherence in clinical research. *Psychiatry Research*, *294*, 113558. https://doi.org/10.1016/j.psychres.2020.113558 |
| [31] | Tucker, C, Behoora, I, Nembhard, H B, Lewis, M, Sterling, N W, & Huang, X (2015). Machine learning classification of medication adherence in patients with movement disorders using non-wearable sensors. *Comput Biol Med.*, *1*(66), 120-134. https://doi.org/10.1016/j.compbiomed.2015.08.012. |
| [27] | Bastidas, O J, Zahia, S, Fuente-Vidal, A, Férez, N S, Noguera, O R, Montane, J, & Garcia-Zapirain, B (2021). Predicting physical exercise adherence in fitness apps using a deep learning approach. *International Journal of Environmental Research and Public Health*, *18*(20), 1-32. https://doi.org/10.3390/ijerph182010769 |
| [14] | Zhou, M, Fukuoka, Y, Goldberg, K, Vittinghoff, E, & Aswani, A (2019). Applying machine learning to predict future adherence to physical activity programs. *BMC Medical Informatics and Decision Making*, *19*(1), 1-11. https://doi.org/10.1186/s12911-019-0890-0 |
