# Supplementary material for: Predicting Adherence to Behavior Change Support Systems Using Machine Learning: Systematic Review
Source: JMIR AI. 2023 Nov 22;2:e46779. doi: 10.2196/46779 (PMC11041458; doi:10.2196/46779)
Supplement: Multimedia Appendix 4 [file ai_v2i1e46779_app4.docx]

Appendix 4. Characteristics of the study and the BCSS.

| Ref. | Aim of the study | Name of BCSS (link/Ref to the BCSS) | Description/purpose of the BCSS | Technology context | User context |
| --- | --- | --- | --- | --- | --- |
| [32] | Use machine learning models to predict user success in achieving set goals in a BCSS | Jellinek digital self-help intervention [40] | Jellinek is an evidenced-based unguided digital self-help intervention for alcohol, cannabis and cocaine use, tobacco smoking, and gambling. It is based on CBT and motivational interviewing techniques and is composed of 6 modules | Web-based | Smoking, alcohol, and illicit drug users |
| [14] | To build predictive models that can identify users in a lifestyle intervention that are at high risk of dropout | LIVA app (app was developed by LIVA healthcare and is available in the iOS store) | The LIVA app is a digital platform designed to facilitate lifestyle changes in users with chronic diseases | Mobile app | Overweight patients and patients with any chronic disease |
| [30] | To distinguish between adherers and non-adherers of BCSS using machine learning algorithms based on user characteristics | Weight and Activity with Blood Pressure Monitoring System (WANDA-B) [41] | WANDA-B is an android-based activity monitor app developed to collect and capture daily weight, blood pressure, heart rate, and symptoms that are difficult for patients to report | Mobile app | Patients with heart failure |
| [34] | To use supervised machine learning to investigate both established and novel predictors for iCBT adherence | Internet-delivered cognitive behavior therapy (iCBT) [42] | iCBT was developed to remotely monitor symptoms of myocardial infarction, depression and anxiety (MI-ANXDEP) | Web-based | Patients with MI-ANXDEP |
| [37] | To demonstrate the feasibility of predicting user dropout from data generated by a BCSS | Sleep Healthy Using the Internet (SHUTi) [31] | SHUTI is a fully automated web-based program that delivers cognitive behavioral therapy (CBT) for insomnia. It is informed by the model for internet interventions and based on the primary principles of face-to-face CBT for insomnia. It contains 7-cores that are dispensed over time. | Web-based | Patients with insomnia |
| [35] | To use machine learning techniques to predict lapses and evaluate the utility of combining both group- and individual-level data to enhance lapse prediction | Ecological momentary assessment (EMA) and Weight Watchers (WW) program [44] | EMA smartphone app and Weight Watchers (WW) program to monitor dietary relapse | Web-based and mobile app | Overweight/obese adults |
| [31] | To predict medication adherence using ensemble learning and deep learning models | Internet-connected smart sharp bin (SSB) [45] | SSB was developed to remotely monitor medication adherence with injection protocols | Sensor system (medication event monitoring system) | Wide range of patients with chronic diseases who self-administer injectable medication at home |
| [36] | To test the accuracy of medication dosing data to predict medication non-adherence. | AiCure app [46] | AiCure is a smartphone-based platform that uses computer vision technology to confirm medication adherence | Mobile-app | Wide range of patients with chronic diseases |
| [33] | To quantitatively evaluate adherence to medication and drug responses among movement disorder patients | Microsoft Kinect | Microsoft Kinect is an off-the-shelf time flight sensor used to collect a variety of human gait measurements from video data | Sensor system | Patients with Parkinson’s disease |
| [29] | To predict the training adherence behavior for a subsample of BCSS users | Mammoth Hunters (MH) fitness app (developed by Mammoth Hunters S.L. and available in the google play store | MH is a smartphone app that provides physical workouts and monitors physical activity adherence | Mobile app | MH app users who want to lose weight or increase muscle mass |
| [15] | To develop and test adherence prediction models using objectively measured physical activity data | Mobile Phone-Based Physical Activity Education program (mPED) [47] | mPED was designed to monitor exercise relapse | Mobile app | Physically inactive women |
