# Supplementary material for: Predicting Adherence to Behavior Change Support Systems Using Machine Learning: Systematic Review
Source: JMIR AI. 2023 Nov 22;2:e46779. doi: 10.2196/46779 (PMC11041458; doi:10.2196/46779)
Supplement: Multimedia Appendix 5 [file ai_v2i1e46779_app5.docx]

Appendix 5. Input data and feature selection approaches

| Ref. | Adherence problem | Type of collected data | Feature selection technique | Potential predictors (features) | Relevant predictors |
| --- | --- | --- | --- | --- | --- |
| [32] | Adherence to digital cognitive behavioral therapy/healthcare interventions | Log data of achieved target goal | Feature engineering (FE) SHAP and Gini feature importance | Num of; logins, forum visits, participation badges, forum posts, thread posts, achievement likes, write agreement, start video, program goal-slowly stop, program goal-stop, the goal of using the BCSS, daily consumption, engagement, no substance use on weekends | The goal of using the BCSS (quitting substance use, initial daily consumption, no substance use on weekends, and engagement with the BCSS |
| [14] |  | Responses from completed modules and patient-reported outcomes measures (PROM) | Wrapper methods - backward and forward selection algorithm (FSA) | Num of providers, gender, age, treatment groups, days on the platform, weeks of inactivity, start BMI, advice received, and messages sent | Provider of the intervention, 2 weeks of inactivity, and the number of advice received from the health coach |
| [30] |  | data from continuous monitoring of user-health characteristics - daily weight, blood pressure, heart rate, and responses to a symptom assessment questionnaire | Filter method (information gain, correlation coefficient) & embedded methods (information gain + ID3) | Having or not having heart failure specialty care and having or not having an implantable cardioverter-defibrillator | Having an implantable cardioverter-defibrillator |
| [34] |  | Automatically extracted linguistic data during user engagement | Wrapper methods -recursive feature elimination, backward FSA | Self-assessed cardiac fear, sex, the number of words, self-assessed general cardiac anxiety, average sentence length, number of mutual words used | Self-assessed cardiac-related fear, sex, and number of words the patient used to answer the first homework assignment (written verbal responses) |
| [37] |  | user logins, automated emails, sleep diaries & trigger events logged in the system | Feature engineering – grid-based search | Handcrafted features (eg, days since last interaction with the system, sleep duration, preferred arising time, the average time needed to complete homework, differences between awake & arise time, the difference between preferred arise time and actual arise time) and clinically important features (login, recorded trigger, nap time, finished homework, precipitating factor) | Handcrafted features – time to complete certain steps in the intervention, time to get out of bed, days since the last interaction with the system |
| [35] | Diet adherence | Triggers of dietary lapses | N/A | Affect, boredom, hunger, fatigue, hours of sleep, cravings, urges, cognitive load, confidence, motivation, socializing, TV, exercise, negative interpersonal interactions, tempting food availability, alcohol, food ads, planning, missed meal/snack | All selected predictors of lapse triggers |
| [31] | Medication adherence | Injection drops history data and timestamps | Filter method - information gain (FSA) | Historic drops, medication frequency, country, and day of the week for the scheduled medication event | Historic drops/drop status (on-time/not on-time) |
| [36] |  | Videos of dosing behavior and timestamps | Embedded methods – tree-based FSA | Demographics, daily values of adherence, adjusted adherence, past adherence, number of interventions, dose delay, dose length, trial length, micro reimbursements, conditions | Past adherence |
| [33] |  | Time-stamped 3D coordinate skeletal data of the patients | Feature engineering | The entire feature space containing 1890 gait-related features (180 positions, velocity and acceleration features +1710 ratio features from enumerative combinations) | Arm features (elbow joints), knee and spine joint locations |
| [29] | Physical activity adherence | Training behavior | Feature engineering | Potential predictors related to training frequency and time spent in each training session | Training frequency |
| [15] |  | Walking steps and physical activity intensity | Feature engineering | Daily steps, goal-achieving percentage, moderate to vigorous-intensity physical activity (MVPA) in the morning, afternoon, and evening | Steps data and physical activity intensity |
